# Combined Effect of Maternal Separation and Early-Life Immune Activation on Brain and Behaviour of Rat Offspring

**DOI:** 10.3390/biom14020197

**Published:** 2024-02-07

**Authors:** Bharti Biswas, Valsamma Eapen, Margaret J. Morris, Nicole M. Jones

**Affiliations:** 1 School of Clinical Medicine, Faculty of Medicine & Health, UNSW Sydney, Kensington, NSW 2052, Australia; bharti.biswas@gmail.com (B.B.); v.eapen@unsw.edu.au (V.E.); 2 School of Biomedical Sciences, Faculty of Medicine & Health, UNSW Sydney, Kensington, NSW 2052, Australia

**Keywords:** maternal separation, LPS, animal model, gene expression, memory, anxiety

## Abstract

Adversity during early life, a critical period for brain development, increases vulnerability and can have a lasting impact on the brain and behaviour of a child. However, the long-term effects of cumulative early-life stressors on brain and behaviour are not well known. We studied a 2-hit rat model of early-life adversity using maternal separation (MS) and immune activation (lipopolysaccharide (LPS)). Rat pups underwent MS for 15 (control) or 180 (MS) minutes per day from postnatal day (P)2–14 and were administered saline or LPS (intraperitoneal) on P3. Open-field (OFT) and object-place recognition tests were performed on rat offspring at P33–35 and P42–50, respectively. The pre-frontal cortex (PFC) and hippocampus were removed at the experimental endpoint (P52–55) for mRNA expression. MS induced anxiety-like behaviour in OFT in male and reduced locomotor activity in both male and female offspring. LPS induced a subtle decline in memory in the object-place recognition test in male offspring. MS increased glial fibrillary acidic protein (GFAP) and brain-derived neurotrophic factor expression in PFC and ionised calcium-binding adapter molecule-1 expression in male hippocampus. MS and LPS resulted in distinct behavioural phenotypes in a sex-specific manner. The combination of MS and LPS had a synergistic effect on the anxiety-like behaviour, locomotor activity, and *GFAP* mRNA expression outcomes.

## 1. Introduction

Early-life exposures to adverse events can be broadly divided into biological (e.g., infection, malnutrition, etc.) or psychological (e.g., neglect, abuse). Exposure to one type of early-life adversity increases the probability (65–90%) of experiencing subsequent adversities [1]. The cumulative stress hypothesis proposes that a combination of stressful events occurring during pregnancy and the early developmental period can lead to increased risk of neurodevelopmental or psychiatric disorders later in life [2,3]. One example of this is the coexistence of extreme poverty and malnutrition during early life increasing vulnerability to brain and behavioural disorders [4]. While human cohort studies have shown that adversity in early life can enhance the risk of hallucinations, anxiety, depression, etc. [4,5] the mechanisms underpinning longer-term consequences require investigation in animal models in relation to potential contributing factors. In order to study the long-term effects of cumulative early-life stress on the brain and behaviour of rats, two types of established interventions were applied in the current study, maternal separation (MS) and immune activation (via lipopolysaccharide (LPS)).

Exposure to multiple adversities during early life can enhance the risk of developing neurodevelopmental and neuropsychiatric disorders. These disorders include intellectual disability (ID), attention-deficit hyperactivity disorder (ADHD), autism spectrum disorder (ASD), schizophrenia, cerebral palsy, and Tourette’s syndrome, among others [2,6]. These disorders are characterised by a wide range of clinical symptoms, including motor and sensory impairment, delayed development of speech and language, difficulties in memory, learning, behaviour, and social interactions, attention deficits, heightened anxiety, and hyperactivity responses [1,7].

The 2-hit hypothesis proposes that an individual is predisposed to the clinical phenotype by a combination of early-acting risk factors, comprising a first ‘hit’ followed by a second ‘hit’ occurring at a later stage of development, resulting in the onset of clinical symptoms. Given the heterogeneous clinical symptoms and complex aetiology of neurodevelopmental disorders (NDDs), we propose to use a 2-hit model, whereby exposing the rats to first hit, maternal separation would enhance the vulnerability and susceptibility to the second hit, LPS administration.

After birth, the absence of tactile stimulation can cause disruption in the mother–infant relationship, impacting the behaviour of a child [3,8]. Thus, to understand the physiological effects of MS on behavioural outcomes in offspring, a well-established MS model in rats was used in the current study. In rats, the early postnatal period (P2–14) is critical for the development of offspring, as it is a peak period of neurogenesis in the brain, and proximity to the mother plays an important role in regulating hypothalamic–pituitary–adrenal axis (HPA) activity, which can mediate stress responses [9,10,11]. MS exposure during this stress hyporesponsive period (first two postnatal weeks in rodents) has been shown to result in higher glucocorticoid receptor expression in the hypothalamus and reduced expression in the hippocampus and pre-frontal cortex (PFC), leading to enhanced cortisol secretion and a delay in termination of the stress response in younger animals. This results in an anxious or hypervigilant state for a longer period than required, impairing stress reactivity, hippocampal-dependent spatial learning and memory, and PFC-dependent tasks such as working memory and cognition [12,13,14,15].

The second intervention studied was immune activation via LPS administration. The immune system is relatively immature at birth; thus, newborns are more sensitive to infection, especially those who are born preterm [16]. In terms of brain development, the P3 rat coincides approximately with the third trimester in human gestation, during which significant brain growth occurs [17]. To better understand some of the structural and behavioural changes due to inflammation in the immature brain, we used LPS, a component of Gram-negative bacterial cell wall, administered on P3 as described previously [18]. Systemic administration of LPS induces a set of changes characterised by temperature change, decreased food and water intake, and reduced locomotor activity known as acute sickness behaviour [19]. LPS activates the central nervous system (CNS), as shown by increased brain levels of interleukins (IL), prostaglandins, and reactive oxygen species (ROS), which can lead to white matter injury (damage in the white matter tracts and myelinated axons, limiting the communication with grey matter areas, neuron cell bodies, glial cells, and dendrites), cerebral palsy, and long-term neurological consequences [18,19,20].

It has been reported that MS-potentiated LPS (administered during the second week of life) induced inflammatory response (higher IL-1B, IL-6 and Tumour Necrosis Factor alpha (TNF-α)) and HPA-axis activation (higher corticosterone level) [21,22]. Wang et al. demonstrated that MS and LPS (administration at P60–66) resulted in more severe depression-like behaviour, higher levels of Nuclear factor kappa B (NF-κB), and lower expression of histone methylation (H3K27me3) in the hippocampus and PFC [23]. While it appears LPS administration exacerbates the effects of MS, limited work has addressed the cumulative impact of combining these interventions during early life. The aim of the current study was to investigate the combined long-term effects of MS and early-life bacterial infection (LPS administration at P3) on the brain and behaviour of rat offspring. The prediction was that MS rats would demonstrate more severe behavioural deficits following LPS exposure and these exaggerated behavioural changes would be driven by heightened neuro-inflammation. To verify the hypothesis, memory, locomotor activity, and anxiety behaviour were studied during early adulthood in both male and female rat offspring. To determine some of the drivers behind the behavioural changes, the expression of glial fibrillary acidic protein (GFAP), ionised calcium-binding adapter molecule-1 (Iba-1) and brain-derived neurotrophic factor (BDNF) were assessed.

## 2. Materials and Methods

**Figure 1:**
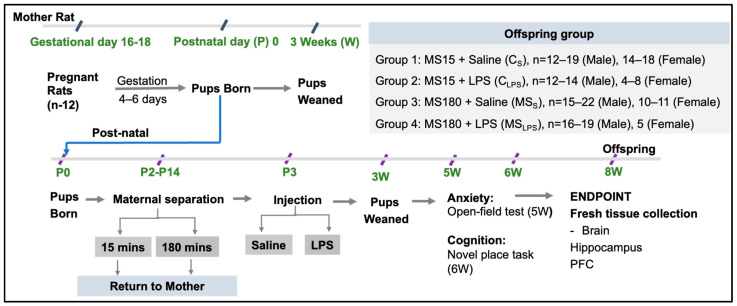
Schematic presentation of the experimental timeline.

All animal experiments were performed in accordance with the Australian National Health and Medical Research Council code of practice and with approval of UNSW Animal Care and Ethics Committee (ACEC No. 20/77A and 20/77B). Sixteen *Sprague-Dawley* pregnant female rats (gestational day (GD) 16–18, 328–425 g) were housed singly, and chow and water were provided ad libitum. After pups were born (P0), litters were adjusted on P1 to 12 pups/litter. All mother rats were monitored daily and were weighed twice weekly. The litter size was adjusted to 12 pups on P1, and on P2, the litters were divided into MS15 and MS180 groups through counterbalancing based on the litter size on P1 (MS15, 14.5 ± 0.6 and MS180, 14.8 ± 0.7 pups) and sex ratios on P2 (MS15, 1.1 ± 0.2 and MS180, 1.4 ± 0.2, Male/Female). Figure 1 represent the Schematic presentation of the experimental timeline. The rats were housed in the Animal Services Facility University of New South Wales (UNSW) Sydney, Australia under standard laboratory conditions of 21 ± 2 °C; 12:12 h light/dark.

### 2.1. Maternal Separation (MS)

On P2, half of the litters were separated from their mothers for 15 min (control group ‘C’) and the other half were separated for 180 min (MS group ‘MS’) [13]. Separated pups were placed in a different room from the mother on a heating pad (30 °C) once/day from P2 to P14 at a similar time (11 a.m.–2 p.m.) each day.

Pups were born (P0) 4–6 days after arrival of pregnant rats. From P2–14, pups were separated from their mothers for either 15 min or 180 min. On P3, saline/LPS (i.p.) was injected into the pups. At P20, pups were weaned. The groups after MS and LPS injection were designated as C_S_, C_LPS_, MS_S_, and MS_LPS_. One week after weaning, mothers were euthanised. Behavioural testing was performed on offspring from 5 to 6 weeks of age. At the age of 8 weeks, rats were euthanised and fresh tissues collected and snap frozen (Figure 1).

### 2.2. LPS Administration

LPS was freshly prepared in sterile saline and administered intraperitoneally (i.p.) at doses of 1 mg/kg, 0.3 mg/kg, and 0.1 mg/kg. On P3, half of the rat offspring were injected with sterile saline (0.9% NaCl solution) i.p. as vehicle, and the other half with LPS [24,25].

After the initial dose of LPS was administered at 1 mg/kg and induced lethality; out of 6, 4 pups died in the first litter. Subsequently, the LPS dose was reduced to 0.3 mg/kg [26,27]. Half of the pups in five litters were injected with 0.3 mg/kg LPS, and the other half received saline. Out of 32, 14 pups died after 0.3 mg/kg LPS administration. LPS dose was further reduced to 0.1 mg/kg [28] in close consultation with the UNSW Animal Ethics Committee. As described in the manuscript, after analysing the anthropometric, behavioural, and mRNA expression data, no effect of different LPS dose was observed on outcome measures. Thus, we feel it is appropriate in line with the principle of reduction of animal usage.

The temperature of the pups was measured through an infrared thermometer. The resultant data were analysed after combining pups of all three LPS doses (1 mg/kg, 0.3 mg/kg, and 0.1 mg/kg). For all outcomes measured, no differences were observed across the three LPS doses of the surviving animals, so combined LPS data are presented. The groups after MS and LPS injection were designated as C_S_, C_LPS_, MS_S_, and MS_LPS_. On P20, offspring were weaned and housed 3–4 rats/cage, according to their treatment group, with male and female offspring housed separately. All rats received a standard rodent chow diet and potable water ad libitum for the remainder of the study.

### 2.3. Behaviour Tests

Previous studies have shown that a combination of stressful events during the early developmental period can lead to increased risk of NDDs later in life. As adolescence or pre-puberty is a critical stage of brain maturation, in the current study, we chose to perform behavioural tests on adolescent rats to determine some of the longer-term outcomes and examine the individual and combined effects of MS and LPS administration. All the behavioural tests were conducted during the light cycle between 10 a.m. and 4 p.m.

### 2.4. Open-Field Test (OFT)

The apparatus consists of a square acrylic arena (69 cm × 69 cm × 49 cm), brighter in the middle (135–140 lux) and darker at the periphery (70–100 lux). The OFT was performed for 10 min. Video recordings made for both the open-field test and object-place recognition test were scored using AnyMaze software v4.96. The test was performed from P35 to 37 with 64 males and 39 females as described in Kendig et al., 2019 [29].

### 2.5. Object-Place Recognition Test

The apparatus for both tests consisted of a square black box (69 × 69 × 49 cm, 30–45 lux), and the objects used for testing were different in shape and material (three sets of each object). The test was performed from P42 to 50 with 64 males and 34 females in a three-day test, divided into three phases [29,30].

(1) Habituation—on the 1st and 2nd days, rats were allowed to become familiar and explore the empty arena for 10 min on each day.

(2) Familiarisation—on the 3rd day, rats were placed in the arena with two identical objects and allowed to explore the arena and the objects for 5 min.

(3) Object-place recognition test—rats were placed back in the arena after 5 min of retention time, with one of the objects moved to a novel location/place and the other object at familiar location, and allowed to explore the objects for 3 min.

The object locations were counterbalanced across the groups. The interaction time spent with both objects in the familiarisation and testing phases was recorded. Exploration of the object at the novel place was defined as the rat sniffing, licking, or intentionally touching the object within a 2 cm radius (excluding the tail or hind limbs touching or jumping on the objects).

The results of the habituation phase were expressed in distance travelled (m), % time active, and mean speed (m/min). The results of the test are expressed as exploration ratio and total exploration time. Total exploration time was the sum of the time spent interacting with both familiar and novel place objects. Exploration ratio was calculated as the time spent interacting with the object at the novel place divided by the total exploration time.

$$\mathrm{Exploration}\;\mathrm{ratio}=\;\frac{\mathrm{Interaction}\;\mathrm{with}\;\mathrm{object}\;\mathrm{at}\;\mathrm{novel}\;\mathrm{place}}{\mathrm{Interaction}\;\mathrm{with}\;\mathrm{object}\;\mathrm{at}\;\mathrm{familiar}\;\mathrm{place}+\mathrm{Interaction}\;\mathrm{with}\;\mathrm{object}\;\mathrm{at}\;\mathrm{novel}\;\mathrm{place}}$$

### 2.6. Tissue Collection

Mother rats were euthanised one week after weaning, and offspring were euthanised from P52 to P55. Following anaesthesia (pentobarbitone sodium (100 mg/kg, i.p.), rats were euthanised by decapitation, and fresh tissues were rapidly collected (PFC and hippocampus), snap frozen in liquid nitrogen, and stored at −80 °C until use. Tissues were powdered using a tissue pulveriser and stored at −80 °C until assays were performed.

### 2.7. Reverse Transcription Quantitative Real-Time PCR (RT-qPCR)

RNA was extracted using the TRIzol (Merck’s Life Science, San Jose, CA, USA) method, and those RNA samples with OD260/280 values between 1.85 and 2.1 were used for the cDNA synthesis reaction [30]. Following RNA isolation, 1.5 μg of RNA was treated with DNase I Amplification Grade (Merck’s Life Science, San Jose, CA, USA) to remove any contaminating genomic DNA. Then, RNA was reverse transcribed to cDNA using a high-capacity reverse transcription kit (Thermo Fisher Scientific Corporation, San Diego, CA, USA) according to the manufacturer’s instructions. The prepared cDNA was later used for PCR applications and stored at −80 °C. mRNA expression in the cDNA samples was quantified by real-time qPCR performed on the Quant Studio 12K Flex (Thermo Fisher Scientific Corporation, San Diego, CA, USA) using TaqMan inventoried gene expression assays for the genes of interest (Thermo Fisher Scientific Corporation, San Diego, CA, USA). The genes of interest were normalised against the geometric mean of the selected housekeeping genes. The housekeeping genes were selected based on previous publications [31]. Analysis of relative expression was performed using the 2^−ΔΔCT^ method normalised to an independent calibrator.

### 2.8. Statistical Analyses 

All statistical analyses were performed on data which were collected in a manner where the experimenter was blinded to the experimental condition. The results are expressed as mean ± SEM. Initially, all data were checked for normality using Shapiro–Wilk normality test and outliers using mean ± 2std. Data were analysed by unpaired Student’s *t*-test, two-way ANOVA, or three-way ANOVA (for body weight and temperature over time) followed by post hoc Tukey’s honestly significant difference (THSD) test.

## 3. Results

### 3.1. Effect of MS on Mother Rats

No significant effect of MS was observed on the body weight over time (Supplementary Table S1) and anthropometric data (Supplementary Table S2) of the mother rats at the end of the experiment. MS dams took ~250% more time to retrieve their first pup (32.7 ± 9.9 s vs. 95.5 ± 7.3 s, *p* = 0.0005, *t* = 5.233) and the entire litter (92.3 ± 8.5 s vs. 246.9 s ± 15.0, *p* = 0.0001, *t* = 8.450) compared to control dams, indicating that the MS paradigm reduced maternal motivation to retrieve their pups.

### 3.2. Effect of MS and LPS Body Weight of Offspring

A total of 6 h after LPS administration, a ~1 °C drop in body temperature was observed in both male and female offspring, which came back to normal after 24 h (Supplementary Figure S1).

**Figure 2:**
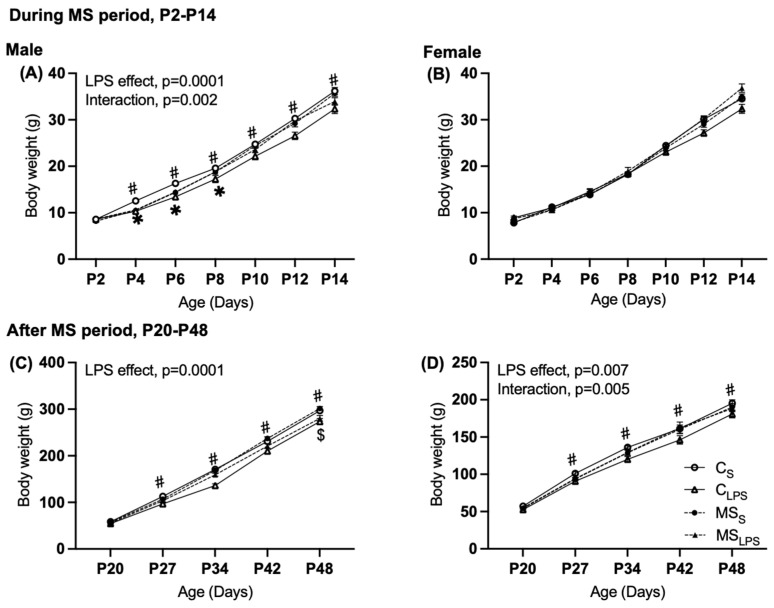
Body weight of male and female offspring (**A**,**B**) during MS period, P2 to P14 and (**C**,**D**) after the MS period, P20 to P48. Data are presented as mean ± SEM and assessed by repeated measures of two-way ANOVA followed by Tukey’s post hoc. C_S_ and C_LPS_ groups are represented by solid lines with open circles (C_S_) or triangles (C_LPS_) and MS_S_ and MS_LPS_ by dotted line with closed circles (MS_S_) or triangles (MS_LPS_). (**A**,**C**) Body weight in male rats (*n* = 14–22), (**A**) overall LPS effect, *p* = 0.0001, and interaction between MS and LPS, *p* = 0.002. (# LPS effect between C_S_ and C_LPS_ *p* = 0.0001, * MS effect between C_S_ and MS_S_ *p* = 0.0001.) (**C**) Overall LPS effect, *p* = 0.0001 (# LPS effect between C_LPS_ and C_S_, *p* = 0.0001, $ LPS effect between MS_S_ and MS_LPS_ *p* = 0.034). (**B**,**D**) Body weight in female rats (*n* = 5–18), (**D**) overall LPS effect, *p* = 0.007, and interaction between MS and LPS, *p* = 0.005 (# LPS effect between C_LPS_ and C_S_ *p* = 0.035).

During the MS period, a main effect of LPS treatment (F(1,70) = 14.349, *p* = 0.0001), and an interaction between MS and LPS treatment (F(1,70) = 10.628, *p* = 0.002) were observed in the body weight of male offspring (Figure 2A). Subsequent analysis revealed that in the saline group, separated offspring were lighter than control (MS_S_ < C_S_ *p* = 0.038, ~5%) and, conversely, in the LPS group, heavier than control (MS_LPS_ > C_LPS_ *p* = 0.016, ~6.7%). In the control (non-separated) group, male LPS offspring were lighter than the saline offspring (C_S_ > C_LPS_ *p* = 0.0001, ~13.5%). In female offspring, no significant effects of MS, LPS, or interaction were observed on body weight (Figure 2B).

After the MS period (P20 to P48), the body weight of male offspring showed a significant main effect of LPS (F(1,70) = 23.563, *p* = 0.0001), whereby LPS offspring were lighter than saline offspring (Figure 2C, by ~9.5%). For female offspring, an overall significant effect of LPS (F(1,38) = 8.092, *p* = 0.007) and an interaction between MS x LPS (F(1,38) = 8.874, *p* = 0.005) were observed in the body weight (Figure 2D). Subsequent analysis revealed that MS offspring were lighter than control in the saline group (MS_S_ < C_S_, *p* = 0.004, ~8.7%) and LPS offspring were lighter than saline in the control group (C_S_ > C_LPS_, *p* = 0.0001, ~15.3%).

### 3.3. Effect of MS and LPS on Anxiety-like Behaviour of Offspring

**Figure 3:**
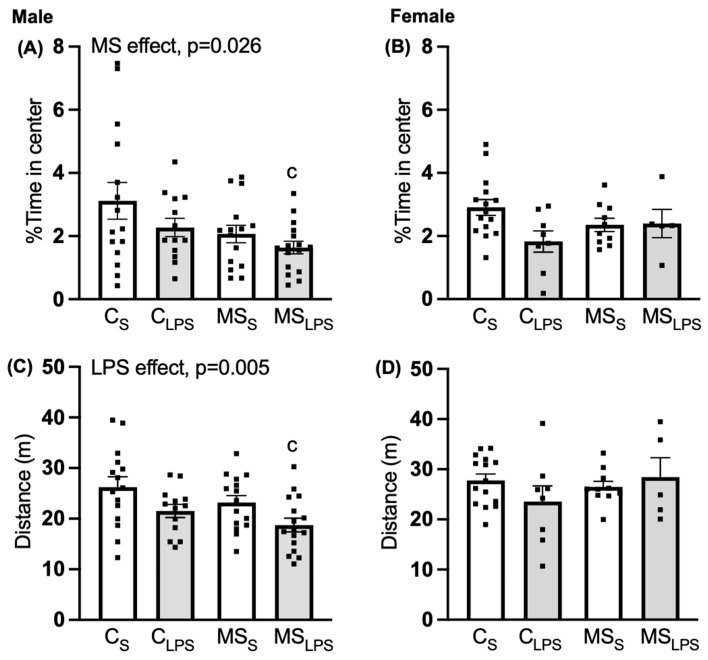
Open-field test at P35–37. Data are presented as mean ±SEM and analysed using two-way ANOVA and Tukey’s post hoc test. Male offspring (*n* = 13–16 per group): (**A**) % time spent in centre, overall MS effect, *p* = 0.026, (^c^ *p* < 0.05 relative to C_S_), (**C**) distance travelled in open field, overall LPS effect, *p* = 0.005, (^c^ *p* < 0.05 relative to C_S_). Female offspring (*n* = 5–15 per group): (**B**) % time spent in centre, (**D**) distance travelled in open field.

In male rats, a main effect of MS was observed in the percent time spent in the centre of the open field (F(1,55) = 5.214, *p* = 0.026, Figure 3A), with the control group spending more time in the centre than the MS group, indicating MS were more anxious than control offspring. For distance travelled, saline-treated rats travelled farther than those treated with LPS (F(1,55) = 8.541, *p* = 0.005, Figure 3C), indicating LPS-treated were less active than saline-treated rats. Further, Tukey’s post hoc analysis revealed a significant difference between C_S_ and MS_LPS_ groups in time spent in the centre (*p* = 0.026) and distance travelled (*p* = 0.005), indicating a synergistic effect of the combined stressors, with enhanced anxiety-like behaviour and reduced locomotor activity in the MS_LPS_ group.

In female rats, no effect of MS or LPS was observed in the percentage time spent in the centre (Figure 3B) and distance covered in the open field (Figure 3D).

### 3.4. Effect of MS and LPS on Locomotor Activity of Offspring

**Figure 4:**
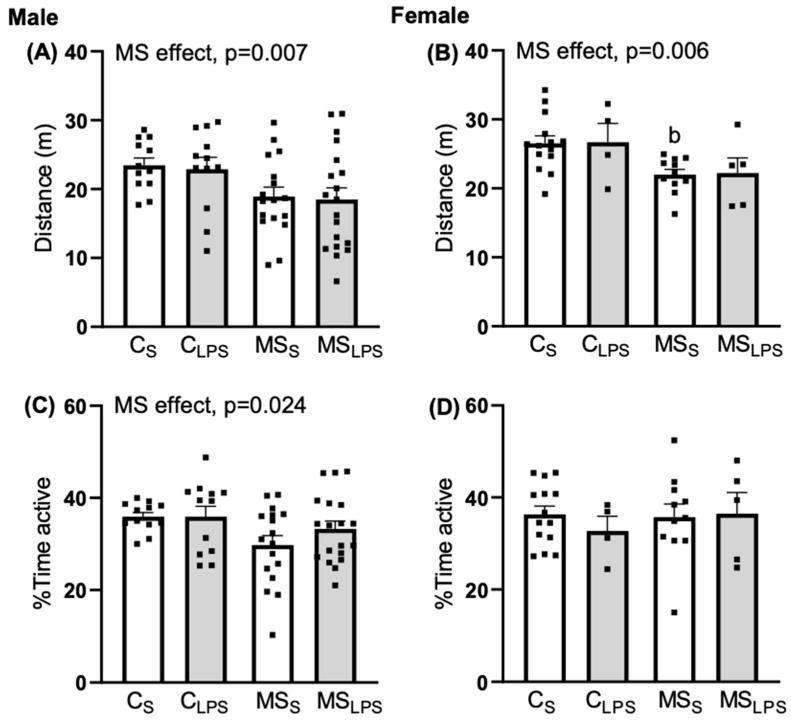
Second day of habituation phase of object-place recognition test at P40–48. Data are presented as mean ± SEM and analysed using two-way ANOVA and Tukey’s post hoc test. Left—male offspring (*n* = 12–19 per group): (**A**) distance travelled, overall MS effect, *p* = 0.007, (**C**) % time active, overall MS effect, *p* = 0.024. Right—female offspring (*n* = 5–14 per group): (**B**) distance travelled, overall MS effect, *p* = 0.006 (^b^ *p* < 0.05 relative to C_S_), (**D**) % time active.

In male rats, a main effect of MS was observed in the distance travelled (F(1,56) = 7.892, *p* = 0.007, Figure 4A), and percentage time active (F(1,56) = 5.344, *p* = 0.024, Figure 4C) on the locomotor activity of the rat offspring. Thus, control offspring covered more distance and were more active than MS offspring. In female rats, an MS effect was observed in distance travelled (F(1,30) = 8.506, *p* = 0.007, Figure 4B) while no effect of MS and LPS was observed in percentage time spent active (Figure 4D). Control female offspring covered more distance than MS offspring. Further, Tukey’s post hoc analysis revealed a significant difference between C_S_ and MS_S_ in distance travelled (*p* = 0.036) and mean speed (*p* = 0.036).

### 3.5. Effect of MS and LPS on Memory of Offspring 

**Figure 5:**
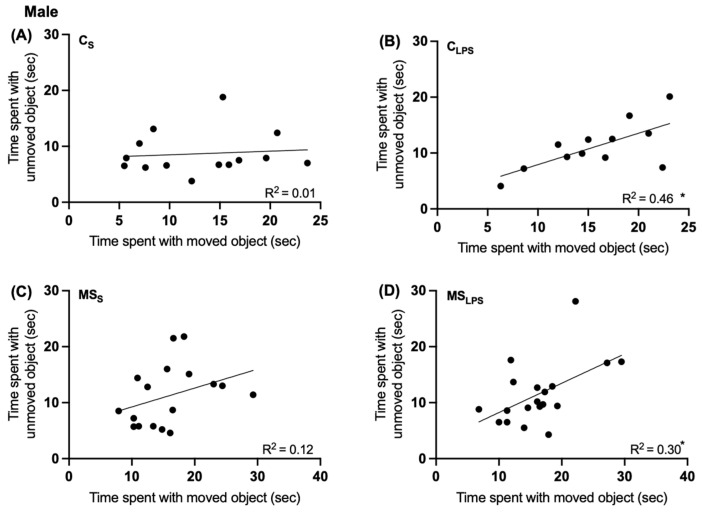
Simple linear regression analysis of time spent interacting with moved and unmoved objects in male offspring at P40–48. Time spent with the unmoved object and time spent with the moved object during place recognition test in male offspring (*n* = 12–19) C_S_ (**A**), C_LPS_ (**B**), MS_S_ (**C**), MS_LPS_ (**D**) groups; (**B**,**D**) * *p* < 0.05.

In both male and female rats, no significant effect of MS or LPS was observed on the total exploration time and exploration ratio between the groups (Supplementary Figure S2) in the object-place recognition test. However, in male rats, a simple linear regression analysis indicated that the C_LPS_ (Figure 5B) and MS_LPS_ (Figure 5D) groups performed the test differently than the C_S_ (Figure 5A) and MS_S_ (Figure 5C) groups. The more time C_LPS_ and MS_LPS_ rats spent with the unmoved object, the more time they spent with the moved object (*p* = 0.0148 and *p* = 0.02, respectively), suggesting that both LPS treatment groups checked the objects continually during the test. This effect was not seen in the C_S_ (*p* = 0.74) and MS_S_ (*p* = 0.17) groups.

### 3.6. Effect of MS and LPS on Anthropometric Data of Offspring at Endpoint (P51–56)

As illustrated in Table 1, in male offspring, a main effect of LPS was observed on body weight, naso-anal length, brain weight, and % brain/body weight. LPS-injected offspring demonstrated reduced body weight, naso-anal length, brain weight, and core and % brain/body weight as compared to saline offspring. A main effect of MS was observed on % brain/body weight in male offspring, whereby MS offspring showed reduced % brain/body weight compared to CS offspring. In female offspring, no effect of MS or LPS was observed on any of the anthropometric data.

### 3.7. Effect of MS and LPS on PFC Gene Expression

**Figure 6:**
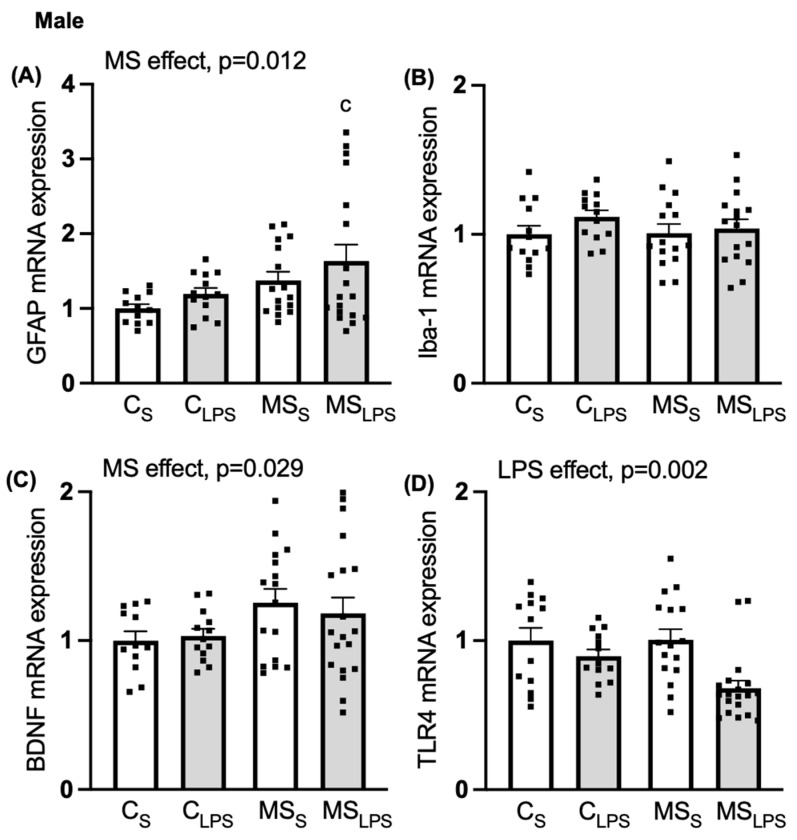
Relative gene expression in PFC in male offspring. Data are shown as mean ± SEM (*n* = 10–18 per group), assessed by two-way ANOVA followed by Tukey’s post hoc. Relative gene expression of (**A**) *GFAP*, overall MS effect, *p* = 0.012 (^c^ *p* < 0.05 relative to C_S_), (**B**) *Iba-1*, (**C**) *BDNF*, overall MS effect, *p* = 0.029, and (**D**) *TLR4*, overall LPS effect, *p* = 0.002.

As the behavioural effects we observed were more pronounced in male compared to female offspring, further analysis of gene expression changes was performed only in male rats (*n* = 65). A main MS effect was observed in *GFAP* (F(1,55) = 6.695, *p* = 0.012, Figure 6A) and *BDNF* expression (F(1,56) = 5.009, *p* = 0.029, Figure 6C), indicating that *GFAP* and *BDNF* expression were increased in the PFC of MS offspring compared to control offspring. Further post hoc analysis of *GFAP* expression revealed a significant difference between C_S_ and MS_LPS_ groups (*p* = 0.03), indicating a synergistic effect of combined MS and LPS in the MS_LPS_ group. A main effect of LPS was observed on *TLR4*, whereby its expression was reduced in LPS compared to the control group (F(1,57) = 10.756, *p* = 0.002, Figure 6D). No effect of MS, LPS, or interaction was observed in *Iba-1* expression in the PFC (Figure 6B).

### 3.8. Effect of MS and LPS on Hippocampus Gene Expression

**Figure 7:**
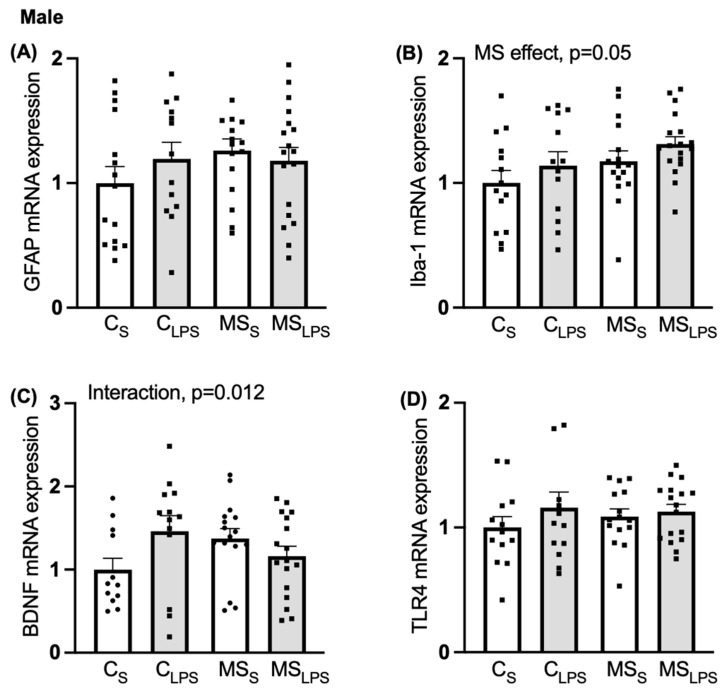
Relative gene expression in the hippocampus in male offspring. Data are shown as mean ± SEM (*n* = 10–18 per group) and assessed by two-way ANOVA followed by Tukey’s post hoc. Relative gene expression of (**A**) *GFAP*, (**B**) *Iba-1*, overall MS effect, *p* = 0.05, (**C**) *BDNF*, interaction between MS and LPS, *p* = 0.012, and (**D**) *TLR4*.

A main MS effect was observed on *Iba-1* expression (F(1,55) = 6.695, *p* = 0.05, Figure 7B), indicating that *Iba-1* expression was enhanced in MS compared to control offspring. In *BDNF* expression, an interaction between MS and LPS was observed (F(1,56) = 5.009, *p* = 0.012, Figure 7C). Further analysis revealed that MS offspring had higher expression of *BDNF* mRNA than control offspring in the saline group (C_S_ vs. MS_S_, *p* = 0.035) and LPS offspring showed higher expression of *BDNF* mRNA than saline offspring in the control group (C_S_ vs. C_LPS_, *p* = 0.016). No significant effect of MS or LPS was observed on *GFAP* (Figure 7A) and *TLR4* (Figure 7D) and expression.

## 4. Discussion

This study confirmed previous observations that MS reduced maternal motivation to retrieve pups back to the nest. LPS reduced the body weight of both male (P2–P48) and female (P20–P48) offspring and induced subtle memory deficits only in male offspring. An interaction between the two stressors, MS and LPS, was observed in the body weight trajectory of pups. A single stressor (either MS or LPS) reduced body weight in male (P2–P14) and female (P20–P48) rats, while the combination of stressors (MS+LPS) mitigated the reduction in body weight. In the offspring, MS induced anxiety-like behaviour in males and reduced locomotor activity in both males and females. MS also increased *GFAP* and *BDNF* expression in PFC and *Iba-1* expression in male hippocampus. No additive effect of combining both MS and LPS stressors was observed in the brain and behavioural outcomes that were measured. MS and LPS resulted in distinct behavioural phenotypes in a sex-specific manner.

### 4.1. Effect of MS on Dam Behaviour 

MS dams showed reduced motivation to retrieve their pups to the nest compared to controls, as evidenced by increased time taken for the task, which is in line with other studies [31,32]. MS has previously been shown to increase depression-like behaviour in mothers, assessed by increased immobility in a forced-swim test [10,33] and decreased sucrose preference [13], which may contribute to reduced motivation in mothers observed in the current study. These studies suggested that long periods of MS can reduce the quality of maternal care, reflected in reduced licking and grooming from the mother after reunion with the offspring [10,13,32].

### 4.2. Effect of MS on Offspring Behaviour 

The OFT results from the current study showed that MS male offspring exhibited increased anxiety-like behaviour and reduced locomotor activity and percentage time active compared to controls. In female offspring, MS reduced locomotor activity only, with no effect observed on anxiety or percentage time active, which may be related to the smaller number of females in the cohort. A similar finding of enhanced anxiety in both male and female rats following MS was reported by many researchers when assessed by OFT or elevated plus maze (EPM—another anxiety test) [34,35,36,37]. Previous research has also shown that MS or maternal deprivation can lead to altered HPA-axis responsiveness, resulting in enhanced cortisol secretion impairing stress reactivity, which might underpin increased anxiety, depression-like behaviour, and reduced locomotor activity in rodents [38,39,40]. In this study, we observed reduced locomotor activity in both male and female offspring, which is in line with previous studies where decreased locomotor be-haviour was observed due to hypoactivity in MS rats [41,42].

Furthermore, the MS_LPS_ group displayed increased anxiety-like behaviour and reduced locomotor activity, suggesting that MS increased the susceptibility to LPS, leading to worsened response in the combined group.

### 4.3. Effect of MS on PFC and Hippocampal Gene Expression in Male Offspring 

In the hippocampus and PFC, analyses of various inflammatory and glial markers indicated that MS male offspring had higher expression of the microglial marker *Iba-1* (hippocampus) and the astrocytic marker *GFAP* (PFC) compared to controls. These outcomes support previous findings where MS enhanced glial responses as shown by an increase in *Iba-1* expression in the dentate gyrus of the hippocampus [43], CA1 of the hippocampus and PFC [23], and GFAP in the PFC [44] compared to non-separated adult rats. In rodents, the second postnatal week is characterised by a peak of gliogenesis, hence separation from mothers during this critical time period can interfere with glial maturation [45,46,47], which may relate to the alterations in *GFAP* and *Iba-1* observed in the current study. *Iba-1* expression was shown to be higher in the hippocampus but not PFC of male MS offspring. Banqueri et al. also demonstrated enhanced expression of *Iba-1* in the CA3 region of the hippocampus with no change in the PFC, which is in line with brain region-specific responses to stressful stimuli [45].

Other studies have reported that increased microglial activation or inflammation due to MS exposure in rodents is associated with mood disorders and depressive and anxiety-like behaviour [48,49]. Thus, the increased *GFAP* expression in the PFC and *Iba-1* expression in the hippocampus we observed in response to MS exposure in the present study may have contributed to the behavioural deficits observed, including increased anxiety and reduced locomotor activity.

Moreover, the MS_LPS_ group displayed an increase in *GFAP* mRNA expression, while no effect of MS and LPS was observed, indicating a synergistic effect of the combined stressors. The is similar to the pattern seen in the open-field test, where MS_LPS_ rats showed higher anxiety-like behaviour and reduced locomotor activity. This interpretation supports the 2-hit theory of neurodevelopmental and neuropsychiatric disorders, which posits that exposure to first stressor, MS, enhanced the susceptibility to second stressor, LPS, resulting in worsened response in the combined group, the MS_LPS_ group.

In the PFC, *BDNF* expression was increased in MS offspring, while in the hippocampus, its expression was increased following LPS and MS alone; it would appear that combining these two stressors restored *BDNF* expression back to control levels. BDNF is associated with growth and differentiation of new neurons and synapses [50]. In the case of patients with depression, studies have found decreased hippocampal mRNA expression of *BDNF* [51,52], while in the animal models, previous work showed conflicting results. Some studies have suggested that MS exposure, 24 h maternal deprivation, or stress during gestation can decrease BDNF protein levels in the hippocampus or PFC of adult offspring [53,54,55], while others have found elevated BDNF protein in the hippocampus and no change in the PFC and hypothalamus in three-month-old rats post MS [56]. One study in rats found that MS enhanced *BDNF* mRNA expression at P21 and 2 months, but it is noteworthy that the expression was reduced by 15 months of age [57]. These studies suggest the BDNF response to stress exposure may differ with brain region, in addition to the age of the rat. Further experiments, such as immunohistochemistry or Western blotting, could provide a better understanding of this enhanced mRNA expression of *BDNF* in MS offspring observed in the current study.

### 4.4. Effect of MS and LPS on Body Weight of Offspring 

LPS administration reduced body weight of male rats from P4 to P48 and females from P20 to P48, only in controls, i.e., C_LPS_ < C_S_; this was not observed in MS rats. Previous studies have also shown that neonatal injection of LPS within the first week after birth in rats and mice reduced body weight up until adolescence [58,59,60,61]. It is well established that LPS induces sickness behaviour, which leads to a decrease in food intake. This effect is mediated by increases in several pro-inflammatory cytokines (IL-1, TNF-α, IL-1β, prostaglandin E2 (PGE2), interferon gamma (IFN-γ), and arachidonic acid metabolites), which induce changes in the hypothalamic feeding regulatory centre, which inhibit feeding [18,19,20]. Hence, the decreased body weight in neonates in the current study might be explained by decreased milk consumption due to symptoms of sickness behaviour following LPS administration.

In the current study, a main effect of MS was not observed, although the analysis of interaction between two stressors (MS and LPS) suggested that MS reduced body weight in males during earlier age (P2–14) and after weaning in females (P20–48). There was no additive detrimental effect on body weight when the two stressors were combined (LPS and MS) in either male or female rats. Some authors have reported MS (P2–14, 3 h/day) leads to reduced body weight in both male and female offspring early in life, i.e., until 3 weeks of age [62,63,64]. The reduction in body weight may be explained by the fact that separation from mothers can affect the mother–offspring interaction, leading to inadequate nutrition [65]. Others have proposed that reduced growth hormone in pups resulted in lower body weight and abnormal growth trajectory [66,67,68], similar to our finding where 3 h of MS reduced body weight in offspring until early adulthood.

### 4.5. Effect of LPS on Behaviour of Offspring 

Previous research has demonstrated that neonatal LPS exposure during the first week after birth can lead to memory deficits [18,61,69,70]. Some studies [26,61] have shown that a single LPS exposure ((1 mg/kg) intracerebral injection) in P5 rats leads to memory deficits in adulthood, assessed via passive avoidance test, a hippocampal memory-based test, and locomotion deficits, assessed via vertical activity in OFT. In the current study, early i.p. LPS exposure did not induce frank memory loss, rather rats administered LPS (C_LPS_ and MS_LPS_) performed the task differently. Rats in the LPS groups were able to recognise the object in a novel place, but this involved multiple movements, checking both moved and unmoved objects, compared to saline rats.

A similar subtle change in memory in the object-place recognition test has been reported following a small hippocampal lesion [71]. Subtle memory deficit is associated with slight forgetfulness while carrying out instrumental activities of daily living, without severe memory complaints or subjective cognitive decline in an individual [72]. Among several NDDs, it has been previously reported that schizophrenia is associated with the presence of disruptions in subtle memory such as differentiation between objects. Such disruption in memory has been studied in schizophrenic patients by the Mnemonic Similarity Task (MST), where they tend to mistake similar, but not identical, items more often for ones they studied previously, rather than rejecting them [73].

Although deficits in spatial context memory have been reported in both NDDs (including schizophrenia) and the rodent model of MS, exploring working or executive memory function would have been of interest [10,43]. Unfortunately, this is one of the limitations of the study.

In the current study, a frank memory loss was not observed in male offspring, which might be because rat offspring were exposed to both the stressors (LPS and MS) during early life (from P2 to P14), while previous studies reported that the effects of combined exposure on memory deficits were only observed if rats were rechallenged with LPS at a later age [69,70,74].

In the current study, the effect of LPS on the behaviour of offspring was only observed in male rats, suggesting a sex-dependent effect of LPS. The sex differences in response to the LPS challenge might exist because female rats are more sensitive to bacterial/viral infection during adolescence or adulthood while male rats are more sensitive during early life [75,76]. In addition to infection, there are also sex-dependent differential expressions of neurodevelopmental genes that may also result in sex-dependent differences in the neuronal development, maturation, and consequent developmental trajectory [77,78].

This study’s behavioural and mRNA expression data indicate the presence of inter-individual variability within the same group, suggesting that some animals exhibit greater sensitivity to the stressor compared to others. Inter-individual variability in phenotype generally reflects differences in gene expression among individuals. Both genetic and environmental factors influence many functional or behavioural traits, suggesting the environmental influence over epigenetic modification, which serves as a mechanistic link between genes and the environment [79]. For example, in schizophrenia patients, inter-individual variability has been observed across cognitive tests, giving rise to cognitive heterogeneity as a clinical characteristic of the disorder [80]. A rodent study demonstrated inter-individual variability in social testing environments, attributed to the differential expression of dopamine neurons in response to environmental stimuli [81].

## 5. Conclusions

The combination of MS (P2–14) and LPS (P3) induced a synergistic effect on the anxiety-like behaviour, locomotor activity, and *GFAP* mRNA expression outcomes that were investigated in this manuscript. These results support the 2-hit hypothesis of NDDs and neuropsychiatric disorders, whereby exposure to a first stressor, MS, enhanced the susceptibility to a second stressor, LPS, leading worsened response in the combined MS_LPS_ group.

From the open-field test and object-place recognition test conducted in this study, it can be concluded that MS induced anxiety-like behaviour in male and reduced locomotor activity in both male and female offspring, and LPS administration at P3 induced subtle memory deficits in male offspring. This suggests that the stressors induced distinct sex-specific behavioural phenotypes.

For body weight trajectory, an interaction between the two stressors, MS and LPS, was observed, whereby either MS or LPS reduced the body weight in males (P2–P14) and females (P20–48) while the combination of stressors mitigated the reduction in body weight in the MS_LPS_ group in both males and females. This suggests that exposure to one stressor might lead to resilience to the effects of exposure to the second stressor. Previous research has shown that female offspring from stressed mothers have lower plasma insulin, better homeostatic model assessment for insulin resistance levels (HOMA-IR, an indicator of insulin resistance) [82], and reduced insulin during an oral glucose tolerance test (OGTT) [83] compared to offspring from non-stressed mothers, suggesting there is a programmed resilience in metabolism due to early-life stress. Similarly, in this manuscript, administration of LPS did not lead to a further reduction in MS offspring body weight, suggesting exposure to combined stressors did not lead to additive effects. The exposure to one stressor might lead to resilience to the exposure of the second stressor. Hence, the mitigation in further reduction in body weight might be due to adaptation to exposure to the second stressor.

## 6. Limitations of the Study

1. Limited number of experiments conducted in the study, which includes only two behavioural tests (OFT and object-place recognition test) and four gene expression analyses in two brain regions (PFC and hippocampus).
2. To assess the impact of MS and LPS on memory function, exclusively the spatial memory test (object-place recognition test) was conducted. Other cognitive domains including working or recognition memory were not explored in this study.
3. Due to the small sample size of female rats, genes expression analysis was only conducted in the male rats.

## Figures and Tables

**Table 1:** Anthropometric data of offspring at endpoint (8 weeks).

Male Offspring						
	Control		MS		MS Effect	LPS Effect
*n* = 14–22	Saline (C_S_)	LPS (C_LPS_)	Saline (MS_S_)	LPS (MS_LPS_)		
Terminal weight (g)	359.2 ± 7.9	326.5 ± 6.9 ^a^	357.9 ± 4.7	344.5 ± 7.3		F(1,70) = 11.44, *p* = 0.001
Naso-anal length (cm)	23.2 ± 0.2	22.5 ± 0.17 ^a^	23.3 ± 0.09	22.8 ± 0.2		F(1,70) = 12.72, *p* = 0.001
Rpwat (g)	1.1 ± 0.09	1.0 ± 0.09	1.1 ± 0.08	1.0 ± 0.09		
Brain wt (g)	2.1 ± 0.03	1.98 ± 0.02	2.01 ± 0.02	1.97 ± 0.01		F(1,70) = 7.27, *p* = 0.009
% Brain wt/body wt	0.57 ± 0.008	0.61 ± 0.01	0.56 ± 0.007	0.58 ± 0.01	F(1,70) = 5.57, *p* = 0.021	F(1,35) = 4.99, *p* = 0.032
Glucose (mmol·L^−1^)	7.9 ± 0.1	7.7 ± 0.1	7.8 ± 0.09	7.9 ± 0.2		
**Female Offspring**						
	**Control**		**MS**		**MS Effect**	**LPS Effect**
*n* = 5–18	Saline (C_S_)	LPS (C_LPS_)	Saline (MS_S_)	LPS (MS_LPS_)		
Terminal weight (g)	212.7 ± 3.6	203.7 ± 4.1	208.8 ± 5.7	210.1 ± 5.2		
Naso-anal length (cm)	18.8 ± 0.1	18.6 ± 0.3	18.8 ± 0.2	18.8 ± 0.2		
Rpwat (g)	0.4 ± 0.02	0.4 ± 0.02	0.5 ± 0.04	0.4 ± 0.01		
Brain wt (g)	1.84 ± 0.01	1.82 ± 0.02	1.78 ± 0.02	1.8 ± 0.02		
% Brain wt/body wt	0.9 ± 0.01	0.9 ± 0.02	0.9 ± 0.05	0.9 ± 0.02		
Glucose (mmol·L^−1^)	7.3 ± 0.1	7.2 ± 0.2	7.4 ± 0.1	6.9 ± 0.2		

Data expressed as mean ± SEM. Effects of MS and LPS were assessed by two ANOVA followed by Tukey’s post hoc test (*p* < 0.05, (^a^ *p* < 0.05 relative to CS). For male offspring, *n* = 14–22, and for female offspring, *n* = 5–18.

## Data Availability

The data supporting these findings are available from the corresponding authors upon reasonable request.

## Supplementary Materials

The following supporting information can be downloaded at: https://www.mdpi.com/article/10.3390/biom14020197/s1. Table S1: Body weight of female dams allocated to control or MS; Table S2: Anthropometric data of dams at endpoint; Figure S1: Body temperature 6 h and 24 h after LPS administration of male and female offspring; Figure S2: Test phase of novel place recognition test performance P40–48.
