# Supplementary material for: Combined Effect of Maternal Separation and Early-Life Immune Activation on Brain and Behaviour of Rat Offspring
Source: Biomolecules. 2024 Feb 7;14(2):197. doi: 10.3390/biom14020197 (PMC10886936; doi:10.3390/biom14020197)
Supplement: Supplementary file 1 [file biomolecules-14-00197-s001.zip › biomolecules-2751760-supplementary.pdf]

Supplementary Materials

Table S1. Body weight of female dams allocated to control or MS.

Weight (g)	Control	MS
PND1	336±16	328±5
PND4	340±15	335±7
PND7	343±15	343±10
PND10	345±16	350±12
PND13	347±17	353±13
PND20	345±16	349±9

Data are shown as mean ± SEM of control (n = 6) and MS (n = 6) dams.
The effect of MS was assessed by repeated measures two-way ANOVA.

Table S2. Anthropometric data of dams at endpoint.

	Control	MS
Terminal weight (g)	335±16.5	334±6.5
Circumference (cm)	22.8±0.3	22.7±0.1
Naso-anal length (cm)	18.3±0.5	19.1±0.2
Glucose (mmol.l^{-1})	6.1±0.1	6.3±0.1
Liver weight (g)	12.9±0.6	13.2±0.6
% Liver/body weight	3.85±0.08	3.96±0.20
Rpwat (g)	1.49±0.20	1.51±0.20
% Rpwat/body weight	0.45±0.06	0.45±0.03
Tibia length (cm)	3.97±0.02	3.98±0.03

Data are shown as mean±SEM of control (n = 6) and MS (n = 6) group.
Effects of MS were assessed on anthropometric data by unpaired Student's t-test.
Liver score was analysed by Wilcoxon matched pairs signed rank test.

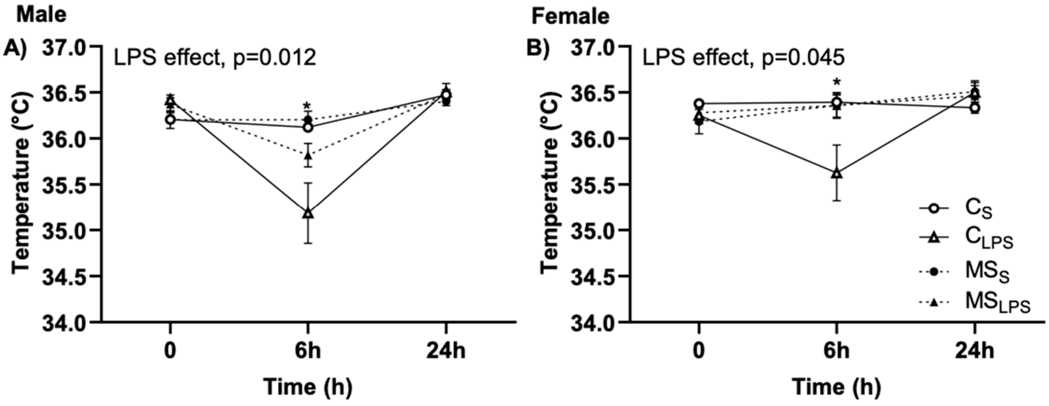

Figure S1. Body temperature 6hrs and 24hrs after LPS administration of male and female offspring**.** Data are presented as mean ± SEM and assessed by repeated measures two-way ANOVA followed by Tukey's post-hoc. (**A**) Body temperature in male rats (n = 13-26) and (**B**) body temperature in female rats (n = 5-18) (*Overall LPS effect at 6hrs, p = 0.0001 for male and p = 0.002 for female rats).

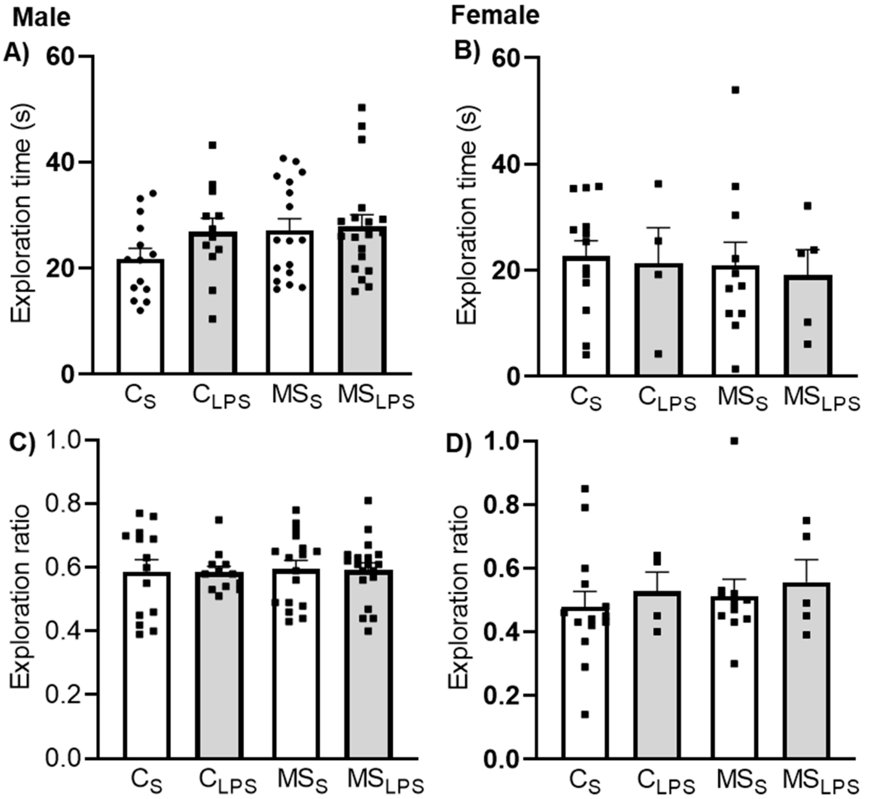

Figure S2. Test phase of object-place recognition test performance P40-48. Data are presented as mean±SEM, analysed using two-way ANOVA, Tukey's post-hoc test. Male offspring (n = 12-19 per group). (**A**) total time spent exploring objects at both novel and familiar places, (**C**) Exploration ratio. Female offspring (n = 5-14 per group), (**B**) total time spent exploring objects at both novel and familiar places, (**D**) Exploration ratio.
